# Zoonotic Hepatitis E Virus: Classification, Animal Reservoirs and Transmission Routes

**DOI:** 10.3390/v8100270

**Published:** 2016-10-03

**Authors:** Virginie Doceul, Eugénie Bagdassarian, Antonin Demange, Nicole Pavio

**Affiliations:** 1 French Agency for Food, Environmental and Occupational Health & Safety (ANSES), Animal Health Laboratory, UMR (joint research unit) 1161 Virology, 94701 Maisons-Alfort, France; virginie.doceul@vet-alfort.fr (V.D.); eugenie.bagdassarian@vet-alfort.fr (E.B.); antonin.demange@vet-alfort.fr (A.D.); 2 French National Institute for Agricultural Research (INRA), UMR (joint research unit) 1161 Virology, 94700 Maisons-Alfort, France; 3 Association of Universities and High Education Institutions (ComUE), Paris-Est Créteil Val-de-Marne University, National Veterinary School, UMR (joint research unit) 1161 Virology, 94700 Maisons-Alfort, France

**Keywords:** hepatitis E virus (HEV), animals, zoonotic reservoir, foodborne transmission

## Abstract

During the past ten years, several new hepatitis E viruses (HEVs) have been identified in various animal species. In parallel, the number of reports of autochthonous hepatitis E in Western countries has increased as well, raising the question of what role these possible animal reservoirs play in human infections. The aim of this review is to present the recent discoveries of animal HEVs and their classification within the Hepeviridae family, their zoonotic and species barrier crossing potential, and possible use as models to study hepatitis E pathogenesis. Lastly, this review describes the transmission pathways identified from animal sources.

## 1. Introduction

Hepatitis E virus (HEV) is a single stranded, positive RNA virus belonging to the Hepeviridae family. Its genome codes for three open reading frames (ORFs) and is 7.2 kb in length. HEV is the leading cause of enterically transmitted hepatitis worldwide. HEV infection can cause an acute hepatitis that is self-limited. However, fulminant hepatic failure can occur in patients with underlying chronic liver disease, in the elderly, and in pregnant women. Complications and extra-hepatic manifestations of hepatitis E, such as acute pancreatitis, renal failure and neurological syndromes including Guillain-Barré syndrome, neuralgic amyotrophy or encephalitis, can also occur [1]. In addition, patients with underlying liver disease and/or immune-deficiencies can develop chronic hepatitis E, exacerbation of liver diseases and cirrhosis, leading to liver transplantation [2,3].

The existence of HEV was postulated for the first time during an outbreak of hepatitis in Kashmir Valley in 1978 [4]. HEV, named at that time “enterically transmitted non-A and non-B hepatitis”, was subsequently identified after a human volunteer was infected experimentally with a pooled faecal extract from affected military personnel [5]. This volunteer later developed acute hepatitis and spherical 27- to 30-nm virus-like particles (VLPs) were visualised in his stool by immune electron microscopy (IEM). In the early 1990s, the HEV genome was cloned and sequenced using samples obtained from experimentally infected macaques [6,7].

**Figure 1:**
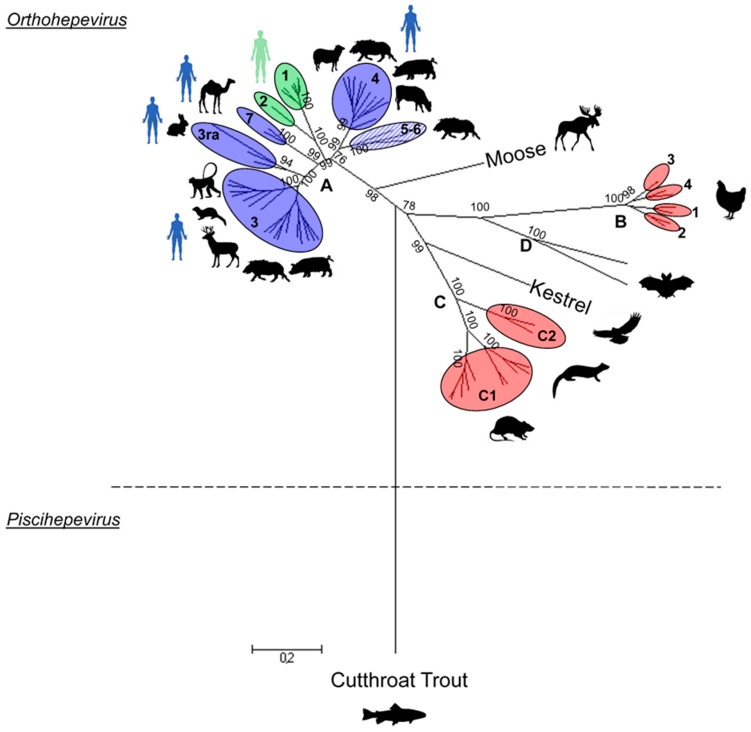
**Phylogenetic tree of representative members of the Hepeviridae family.** The tree was inferred using the Maximum Likelihood method based on the Tamura–Nei model. The analysis was performed with 67 hepatitis E virus (HEV) complete genomes or complete coding sequences available in the GenBank database and representative of each genotype. The sequence size varies between 6543 and 7318 nt in length, and they were aligned using Clustal W. The bootstraps were obtained from 1000 replicates and values over 70% are indicated at the genotype level. The initial tree was obtained by applying the Neighbour-Joining method to a matrix of pairwise distances estimated using the Maximum Composite Likelihood (MCL) approach. The tree is drawn to scale, with branch lengths proportional to the number of substitutions per site. Evolutionary analyses were conducted using Molecular Evolutionary Genetics Analysis (Version 6.0). The *Orthohepevirus* species taxon name is added at the junction of the last common ancestor for each species. Genotypes of non-zoonotic HEV species (red), genotypes including HEV strains isolated from animals and human (blue), genotypes infecting human only (green) and genotypes infecting wild boar that are not linked to human infections (striped blue) are shown.

Since the end of the 1990s, additional HEV-related agents have been identified in a large variety of animals ranging from domestic swine, wild boar, deer, rabbit, mongoose, ferret, rat and chicken to bat and cutthroat trout. Following the identification of these novel strains, a new classification has been proposed that divides the Hepeviridae family into two genera: *Orthohepevirus* and *Piscihepevirus* [8]. Most of the HEV strains identified so far belong to the *Orthohepevirus* genus that is divided into four species: *Orthohepevirus* *A*, *B*, *C* and *D* (Figure 1). Four main genotypes of HEV that belong to the *Orthohepevirus A* species are able to infect humans (HEV-1 to -4). Genotypes 1 and 2 (HEV-1 and HEV-2) infect only humans and are associated with large waterborne epidemics in tropical and subtropical areas. Genotypes 3 and 4 (HEV-3 and HEV-4) are present in humans and other animals, and are the main cause of autochthonous cases of hepatitis E in industrialized countries.

The first HEV-related agent identified in an animal was swine HEV. HEV RNA and HEV-specific antibodies were first detected in domestic swine in Nepal in 1995 [9]. Two years later, a swine strain of HEV was identified in pig herds from the United States (US) and characterised genetically [10]. Pigs inoculated intravenously with swine HEV developed viremia prior to seroconversion, had histological evidence of hepatitis, but did not display clinical symptoms [11]. Swine is the major reservoir of zoonotic HEV-3 and HEV-4 worldwide and is highly prevalent in pig herds. Indeed, anti-HEV antibodies were detected in 46%–100% of swine farms from many countries [12,13,14]. HEV-3 and HEV-4 are also able to infect wild boars, which represent, along with domestic pigs, a major reservoir of zoonotic HEV [12,13]. HEV-3 strains have also been detected in different species of deer and in the Japanese mongoose [15,16,17]. In addition, rabbit HEV-3 strains have been identified in farmed rabbits in China [18] and the US [19], in farmed and wild rabbits in France [20] and also in a pet house rabbit [21]. In the *Orthohepevirus A* species, two other strains of HEV, classified as genotype 5 and 6 (HEV-5 and HEV-6), have also been identified in wild boar in Japan [8,22] and more recently, HEV-7 has been detected in faecal samples from camels [23]. An HEV strain has been characterised in Swedish moose [24] but it is still not assigned to any HEV species.

Additional animal species infected with HEV have also been described. However, the HEV strains detected in these animals are genetically more distant from human HEV strains and are classified as different *Orthohepevirus* species. Avian HEV (*Orthohepevirus B*) was first described in the United States, and is associated with hepatitis-splenomegaly (HS) syndrome in chickens [25], also called big liver and spleen disease [26]. Avian HEV is enzootic in chicken flocks in the US with a seroprevalence of 71% [27]. A rat strain of HEV has been identified in rats [28], with a seroprevalence rate varying from 13% to 90% in many countries [13]. Other HEV variants have been identified in ferrets in the Netherlands [29] and in mink in Denmark [30] (*Orthohepevirus C*). Partial sequences with the highest homology to rat HEV have also been detected in foxes [31]. Another species of HEV was identified in different bats from Central America, Africa and Europe (*Orthohepevirus D*) [32]. Very recently, a HEV strain that might represent a novel *Orthohepevirus* species has been found and characterized in kestrels and falcons in Europe [33]. Finally, a more distant strain of HEV has been discovered in the cutthroat trout in the US and assigned to the *Piscihepevirus* genus [34].

Anti-HEV antibodies have also been detected in different animal species including goats, sheep, buffalo, work horses, cats and dogs. This suggests that these animal species have been exposed to HEV or a closely related agent. However, no HEV RNA has been identified formally in these animals yet. The design of molecular tools used to detect HEV RNA is based on known HEV sequences and might not be able to detect distantly related variants. It is then likely that other animal strains of HEV exist.

In the past 20 years, new molecular tools and the use of metagenomics have highlighted the diversity of HEV strains and susceptible hosts existing. These discoveries have greatly contributed to a better phylogenetic analysis and classification of the Hepeviridae family. In parallel, animal models of HEV infection have been developed and used to study cross-species transmission of the virus and routes of transmission of zoonotic HEV have been identified. This article reviews these recent advances that have contributed to a better understanding of the origins and transmission of zoonotic hepatitis E.

## 2. HEV Phylogeny

To date, 240 complete genomes are available in Genbank (NCBI database), compared to 49 complete genomes available in 2006, with more than 120 sequences of HEV-3. The use of full-length sequences has improved the phylogeny and classification of HEV, but trees generated previously, using shorter sequences from selected genomic regions (partial-ORF1 or partial-ORF2), show similar structures than those generated with full genomes [35].

**Figure 2:**
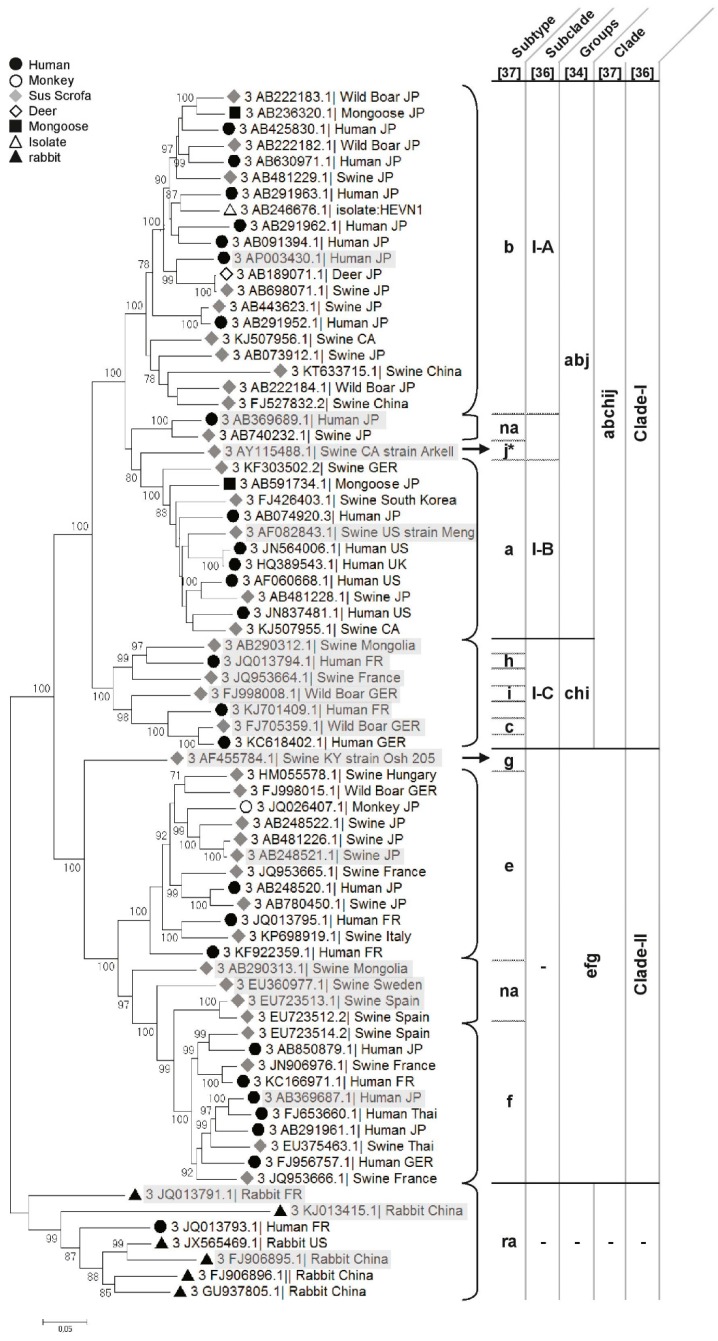
**Phylogenetic tree of HEV-3.** The tree was inferred using the Maximum Likelihood method based on the Tamura–Nei model. The analysis involved the 75 most representative HEV-3 complete sequences/cds available on the GenBank database and aligned using the clustal W method. The bootstraps were obtained from 1000 replicates and values >70% are indicated. The initial tree was obtained by applying the Neighbour-Joining method to a matrix of pairwise distances estimated using the MCL approach. The tree is drawn to scale, with branch lengths proportional to the number of substitutions per site. Evolutionary analyses were conducted using Molecular Evolutionary Genetics Analysis (Version 6.0). Cluster names from the classification proposed by Lu et al., Vina-Rodrigues et al. and Smith et al. (letters a to j and ra cluster) are indicated in the table on the right side of the tree [35,38,39] and by Mirazo et al. (Clade-I and -II and subclades I-A to I-C) [37]. Reference sequences of the different HEV subtypes used by Smith et al. [38] are highlighted in grey. The symbols to the left of the different HEV strains indicate the host of origin. na = non assigned.

Within genotypes, a high diversity can be observed requiring further classification into subtypes or clade/group and subclade/subgroup (genotype 3, Figure 2) [35,36,37]. Thus, a methodology was recently proposed to standardise HEV subtyping. Using this method, subtypes can be redefined when novel full-sequences are added (Figure 2) [38].

### 2.1. Piscihepevirus Genus

The *Piscihepevirus* genus (Figure 1) is composed of one species, *Piscihepevirus A*, and one member, the cutthroat trout virus (CTV) [34] that infects the salmonid fish. CTV is the genetically most distant virus within the Hepeviridae family. It shares 41% to 46% nucleotides (nt) identity and 13% to 26% amino acid (aa) identity with the *Orthohepevirus* genus [8,34].

### 2.2. Orthohepevirus Genus

#### 2.2.1. *Orthohepevirus A*

The *Orthohepevirus A* is the best-characterized species sharing 52.44% to 59.11% nt identity with the other *Orthohepevirus* species, excluding the moose strain (64.5%).

##### Genotypes 1 and 2

These two human genotypes are genetically close with almost 76% nt identity. HEV-1 is well described and divided into six subtypes (1a to 1f) while HEV-2 is less documented and divided into 2 subtypes (2a and 2b) (Figure 1) [38]. Strains belonging to genotype 1 share 88.53% to 94.05% nt identity and are found mainly in Asia, Africa and Mexico [40]. Genotype 2 strains were isolated in Central America (Mexico) and Africa (Tchad, Nigeria) [39,41,42,43].

##### Genotype 3

HEV-3 is the best described and documented genotype in Genbank. Most sequences originate from humans, pigs and wild boars (Figure 2). HEV-3 is divided into 10 subtypes (a to j) and two clades (3abchij and 3efg) [35,36,38,44,45] (Figure 2), sharing 78.74% to 82.46% nt identity. HEV-3 includes some unassigned strains [38] and rabbit HEV for which genotyping it is still under consideration.

Evolutionary studies suggest that the most recent common ancestor for genotype 3 appeared in early 19th century [46] or even in the late 18th century, considering HEV-like outbreak descriptions [47]. Phylogenetic trees are constructed with recent HEV strains using Bayesian approaches to estimate HEV evolution [46]; there are potentially recent bottlenecks through which the various genotypes have passed. A wider sampling of HEV genotypes may show that the present estimates are underestimates of the true evolutionary history of HEV.

1. ***Clade 3abchij***: Within this first clade, HEV strains can be separated into two subclades, 3abj and 3chi, sharing 81.16% to 85.33% nt identity (Figure 2) [35]. HEV strains that cluster in the 3chi subclade share 84.7% to 96.46% nt identity and originate from Europe (France and Germany) and Mongolia. HEV strains within the 3abj subclade share more than 83.75% nt identity. They were isolated in Asia, Europe and North America and are predominantly circulating in Asia and North America. Complete genomes of subtype 3a are from North America and evolutionary studies suggest that it came from Asia and diverged from subtype 3b to subtype 3a in the early 1920s [37,40]. In the subtype 3b, nearly 90% of the full genomes are from Japan, sharing more than 95% nt identity [35,37] (Figure 2). Studies on the origin of HEV in Japan suggest that HEV-3 was imported from Europe in the early 20th century and then diverged into the 3b subtype [37,40]. The subtype 3j was isolated from a pool of pig faecal samples in North America [48], thus, full genome sequences from single animals must be added to validate this subtype [35].
2. ***Clade 3efg:*** This clade includes the three subtypes e, f and g, sharing 82.75%–90.57% nt identity, and 3 non-assigned subtypes. Subtypes 3e and 3f are mainly found in Asia and Europe [49,50]. Evolutionary studies have hypothesised that these HEV strains have emerged in Europe around 1871 [37]. There is only one complete sequence for the subtype 3g from Kirgizstan, which is the most divergent virus of this clade (Figure 2). The classification of the subtype 3d is based on one partial ORF2 sequence from Taiwan, it shares 86.18% and 84.87% nt identity with the subtypes 3g and 3h, respectively. Subtype 3d does not belong to any clade so far.
3. ***Subtype 3ra (rabbit):*** These strains share 73% to 80% nt identity with other HEV-3 subtypes and form a distinct clade within genotype 3 (Figure 2). This divergence is mainly due to numerous substitutions and insertions in the rabbit HEV genome compared to the other *Orthohepevirus* A HEV strains [8,51]. As rabbit strains better cluster with other genotype 3 strains, they are provisionally assigned as subtype 3ra [8,38] and divided into 2 subclades [38]. This subtype includes a strain isolated from a human case of hepatitis E in France that shares 80.12% to 86.14% nt identity with the other rabbit strains [51].

##### Genotype 4

HEV-4 is mainly found in Asian countries and share between 71.79% and 77.38% nt identity with other genotypes (Figure 1). It is divided into nine subtypes (a–i) mainly isolated from pig, wild boar and human. HEV-4 was also detected in other animals such as sheep, cow and goat in China [52,53]. Nevertheless, more investigations are necessary to determine if these species are reservoirs of HEV-4 or accidental hosts.

##### Genotypes 5 and 6

Genotypes 5 and 6 were amplified from wild boars only. They are assigned as subtypes 5a and 6a (Figure 1), sharing more than 78% nt identity amongst themselves and 71.58%–77.38% nt identity with other genotypes. Up to now, there is no human infection associated with these genotypes.

##### Genotype 7

Three complete or partial sequences assigned to genotype 7 have been described. Two of them were isolated from camel (Figure 1) [23]. The third one originates from a human transplant patient and is lacking most of the ORF3 region [54]. These strains are close to each other (>86% nt identity) and share 72.55%–76.13% nt identity with other genotypes.

#### 2.2.2. *Orthohepevirus B*

*Orthohepevirus B* strains were amplified from chicken and represent the shortest HEV genome (6.65 kb). This HEV species shares 51.47%–55.05% nt identity with other *Orthohepevirus* species. To date, there are four different genotypes described from different countries worldwide [55,56,57] (Figure 1) and sharing a low divergence (<6%) [8].

#### 2.2.3. *Orthohepevirus C*

The *Orthohepevirus C* species shares 51.68%–60.57% nt identity with other *Orthohepevirus* species. Two different genotypes can be distinguished: C1 and C2. Genotype C1 includes strains isolated from rat (Figure 1). However, some variants with incomplete sequences, isolated from bandicoot and Asian musk shrew, cluster also in this genotype [58,59]. The phylogeny analysis of the full genomes available shows three different clusters within this genotype that may constitute three possible subtypes (Figure 1).

Genotype C2 is composed of HEV variants isolated from ferret and mink [8,30] (Figure 1). Only two full sequences of ferret are available sharing 81.9% nt identity.

#### 2.2.4. *Orthohepevirus D*

The *Orthohepevirus D* species includes only bat HEV. To date, three full genomes are available and constitute the shortest mammalian HEV genome (6.8 kb) [32]. This HEV species shares between 52.8% and 56.06% nt identity with other *Orthohepevirus* species (Figure 1). A phylogeny analysis with partial sequences isolated from many different countries has shown a high diversity within the *Orthohepevirus D* species, suggesting that several genotypes can be distinguished [32].

#### 2.2.5. *Unassigned Orthohepeviruses*

##### Swedish Moose

HEV strains were recently isolated from Swedish moose [60] (Figure 1). Only one partial genome with complete ORFs is available. A phylogenetic analysis with partial sequences has shown high similarities between different moose strains from the same geographic region (>91% nt identity) [24]. They form a distinct group within the *Orthohepevirus* genus and are close to the *Orthohepevirus A* species (63% nt identity).

##### Kestrel (*Falconidae*)

Very recently, a full HEV genome was retrieved from Kestrel [33]. This HEV strain is similar to the other HEV strains in terms of genome length and organization with 51.47%–60.57% nt identity with others *Orthohepevirus* species (Figure 1). This cluster could constitute a new *Orthohepevirus* species, close to the *Orthohepevirus C* species (58.65%–60.57% nt identity). Partial sequences show a low diversity (>87% nt and 99% aa identities) but broader investigations are necessary to better characterize it.

Zoonotic strains of the *Orthohepevirus A* species are more frequently studied and better classified than non-zoonotic HEV. Additional HEV complete sequences from ferret, bat, moose or Cutthroat trout, from various origins (geographic, related host), would improve the classification [24,36,61].

## 3. Animal models of HEV

### 3.1. Non-Human Primates (Historical Model)

A number of non-human primate species has shown susceptibility to HEV infection, including chimpanzee, rhesus monkeys, African green monkeys, owl monkey, Tamarin and squirrel monkeys [62]. Natural infection and transmission of HEV-3 has been described in a monkey facility in Japan [63]. In addition, cynomolgus and rhesus macaques can be infected experimentally with HEV-1 to HEV-4 [64,65,66,67] and have served as the primary model of HEV infection [62,68]. Experimental infection of a cynomolgus macaque with a suspension of stool from human patients led to the excretion of VLPs [5,69,70] and the development of hepatitis, characterised by liver enzyme elevations, viremia and seroconversion [69,70,71,72,73]. Moreover, the course of infection in experimentally-infected primates is similar to the one in humans with variable incubation periods. An important application of non-human primate studies was to evaluate the efficacy of potential HEV vaccines [74,75]. Non-human primates were also used to evaluate the zoonotic potential of different HEV strains. It was shown that rhesus monkeys and a chimpanzee, experimentally inoculated with swine HEV-3, developed hepatitis [76]. Inoculation of rhesus monkeys with swine HEV-4 also led to seroconversion and viremia but no significant increase in the serum level of alanine aminotransferase (ALT) was observed [77]. The infection of two cynomolgus macaques with rabbit HEV led to the development of a typical hepatitis, suggesting that rabbits may be a source of human HEV infection [78]. However, attempts of cross-species transmission of avian, rat or ferret HEV to non-human primates under experimental condition were unsuccessful [55,79]. Due to limited resources, ethical concerns, and difficult and expensive experimental procedures, little has been learned about the pathogenesis of HEV using primate models.

The discovery of HEV strains in different animal species has then led to the development of other naturally occurring animal models.

### 3.2. Swine

Swine HEV was identified in 1997 and was shown to be antigenically and genetically related to human HEV [10]. Swine is a natural host of HEV-3 and -4 and specific pathogen free (SPF) pigs have been successfully infected intravenously with samples recovered from patients suffering from hepatitis E infection (HEV-3 and -4) [11,76,80,81]. Infected pigs presented mild gross and microscopic liver lesions, viremia, seroconverted and excreted viable HEV in the faeces. Evidences of extrahepatic sites of HEV replication have also been demonstrated in pigs inoculated intravenously [82,83]. Cross-species transmission experiments were performed using the swine model. SPF pigs were successfully infected with two different rabbit strains of HEV, but not with rat HEV [84]. More recently, transmission of HEV from infected wild boar to wild boar and domestic pigs by contact between the animals was demonstrated [85,86]. However, pigs are resistant to experimental infection with HEV-1 and -2 [81] and swine HEV causes only subclinical infection. There is no evidence of clinical disease or elevation of the liver enzyme ALT in this model, thus limiting its usefulness in pathogenicity studies. Nevertheless, this naturally occurring swine model remains very useful for the study of HEV replication and cross-species infection.

### 3.3. Chickens

A chicken model of HEV infection has been developed that presents some advantages: first, like swine HEV, avian HEV is genetically and antigenically related to human HEV [26]. The genomic organisation is very similar to mammalian HEVs. Moreover, avian HEV can be associated with a hepatic disease (HS syndrome). However, in the field, cases of avian HEV infection are mainly subclinical and the pathogenicity linked to avian HEV does not seem to be strain-dependent [87]. SPF chickens can be readily infected by the natural faecal oral route [88] and mild gross pathological lesions and microscopic liver lesions characteristics of HS syndrome have been observed, making it a homologous animal model system to study HEV pathogenesis and replication. Extrahepatic sites of HEV replication were also identified [89]. Cross-species transmission of chicken HEV to turkeys was demonstrated [90]. Infectious cDNA clones of avian HEV were also constructed and capped RNA transcript were used to infect SPF chickens [91,92,93,94], allowing to study in vivo the role of particular regions of the HEV genome in viral replication and pathogenesis.

### 3.4. Rabbits

Infection of SPF rabbits with rabbit HEV induces virus shedding in faeces, viremia and the development of hepatitis, characterised by histopathological changes and an increase in the level of ALT in the serum [95,96,97]. In addition, chronic hepatitis, characterised by liver inflammation and some degree of fibrosis, was observed in rabbits experimentally infected with rabbit HEV [95]. HEV antigen and RNA were found in extrahepatic tissues in infected rabbits [98] and high mortality and vertical transmission of HEV in pregnant rabbits was demonstrated [99]. However, similarly to the swine model, rabbit HEV induces only a subclinical infection with little or no sign of disease. The rabbit model may be useful to study HEV infection and pathogenesis caused by the rabbit strain of HEV and for vaccine evaluation [95]. Experimental infection of rabbits with human HEV genotype 1 or 4 led to the development of hepatitis in none of the rabbits inoculated with HEV-1 and in seven out of nine rabbits inoculated with HEV-4 although most of the inoculated rabbits seroconverted [97]. Rabbits were successfully infected with swine HEV-4 [61] and SPF rabbits farmed in the same enclosed space as HEV-infected pigs seroconverted [100].

### 3.5. Rats

Rodents have been widely used as animal models in scientific and medical research into parasitic, bacterial and viral diseases. Indeed, rodents are easy to handle, manipulate, house and can be used in great numbers. Infection of Wistar rats (via the intravenous or faecal–oral route) with HEV derived from wild rats, can lead to seroconversion and excretion of rat HEV in stool [101]. However, no change in weight and liver enzyme level was observed. The inoculation of Wistar rats with a human stool suspension known to contain HEV-1, led to a successful infection characterised by virus shedding in the faeces, viremia and histopathological changes in the liver, spleen and lymph nodes [102]. However, in two other reports, the injection of HEV-1, -2, -3 (swine) and -4 (wild boar) failed to induce an efficient infection in Sprague-Dawley or Wistar rats [101,103]. Rats were also inoculated with different RNA transcripts from infectious cDNA clones of rat HEV [104], HEV-4 [105] and swine HEV-3 [106] leading to successful infections. Moreover, attempts to infect Wistar and nude rats with ferret HEV failed [78]. The utility of rats as a model of HEV infection still remains to be demonstrated.

### 3.6. Ferrets

Very recently, two ferrets were inoculated orally with ferret HEV [107], leading to a successful infection characterised by the detection of viral RNA in the stool and the sera, seroconversion and a significant elevation of the liver enzyme ALT. These findings indicate that ferret HEV infection can induce liver damage and ultimately acute hepatitis in ferrets. This suggests that ferrets can be used as a potential animal model to study HEV infection. However, strains naturally infecting this species are close to rat HEV and distant from viruses infecting humans and their use might not be adapted to test antivirals or vaccines.

The limited availability, difficulties in handling, manipulating, housing and the cost of both primates and swine severely restrict their use in large number in research. Moreover, naturally occurring small animal models have shown limits in their use to understand HEV pathogenesis and transmission using human strains. Efforts have then been made to develop alternative small animal models that are not natural hosts of HEV.

### 3.7. Mongolian Gerbils

Mongolian gerbil (*Meriones unguiculatus*) is a common experimental gerbil species that was also suggested as an alternative animal model to study HEV replication and pathogenesis. Indeed, Mongolian gerbils have been successfully infected via the intraperitonal route with a HEV-4 strain recovered from a swine liver sample [108,109]. In addition to viremia and faecal virus shedding, the virus was detected in the liver, kidney and spleen as well as the small intestine. Moreover, characteristic histopathological changes observed in the liver of infected gerbils were similar to those reported in humans, and the liver enzymes ALT, aspartate transaminase (AST) and bilirubin levels in the sera were significantly increased. Finally, HEV RNA was detected in the liver from seven to 42 days post infection, which is consistent with the last days of HEV RNA detection in the swine model [110], suggesting that HEV RNA replication in the Mongolian gerbil is similar to its replication in the swine model. Using this model, a study has also shown that swine HEV-4 is able to cross the blood–brain barrier and replicate in the brain and the spinal cord after experimental infection [111]. Mongolian gerbils could then be useful to study the neurological disorders associated with HEV infection. In addition, a successful infection of Mongolian gerbils with a human HEV-1 strain isolated from an acute hepatitis E patient has been obtained [112]. HEV RNA was detected in the faeces of the infected gerbils and histopathological changes in the liver, spleen and kidney were reported as well as fatigue and hair loss. Mongolian gerbils seem to be a promising model to study HEV-1 and -4 infection and pathogenesis.

### 3.8. Human Liver Chimeric Mice

Mouse is a small animal model that is used as a model for many viral infections. The first attempt to infect C57BL/6 mice with HEV-1, HEV-3 and HEV-4 strains failed [113]. In another study, balb/c nude mice were inoculated with swine HEV-4 and HEV antigens were detected in the liver as well as in different extrahepatic organs. Moreover, histopathological changes in the liver and the spleen and increased levels of liver enzymes were observed [114]. However, as reported for pigs, the inoculated mice showed no clinical signs of HEV infection. Very recently, human liver chimeric mice were developed [115]. The liver of UPA/SCID mice was repopulated with primary human hepatocytes and the animals were inoculated with stool-derived virions from humans infected with HEV-1 or -3. Viremia and faecal excretion were reported. Moreover, the co-housing of an HEV-infected mouse with three naïve humanized mice led to successful HEV infection, demonstrating that HEV infection can be transmitted through the faecal oral route in humanized mice, direct physical contact or micro-injuries [115]. HEV-inoculated human liver chimeric mice were also shown to develop chronic HEV infection [116,117] and the treatment of HEV-infected humanised mice with ribavirin led to a statistically significant decrease in the level of HEV RNA in the serum and the faeces and in no more liver damage [115,116]. The human liver chimeric mouse model seems then to be a valuable tool to study the biology of chronic HEV infection and evaluate preclinical drugs. However, this model does not allow immunopathogenesis studies involving adaptive immune responses. Further refinements, such as the transfer of immune cells, may in part overcome these limitations in the future.

Much effort has then been made recently to develop small animal models to study HEV pathogenesis and inter-species transmission. Mongolian gerbils seem to be a promising model that is easy to handle, cost-effective and can mimic hepatic diseases. However, more studies need to be performed to determine whether this species is susceptible to other genotypes of HEV such as HEV-3 and HEV-7. It is also not clear whether gerbils can be infected via the faecal–oral route and whether transmission studies can be performed in this model. Naturally occurring models such as ferrets could also represent a good alternative. Nevertheless, their susceptibility to human and other HEV strains from the *Orthohepevirus A* species remains to be determined. The identification of new strains and hosts of HEV might help in the near future to the development of a suitable naturally occurring animal model.

## 4. Inter-Species Transmission of HEV

**Figure 3:**
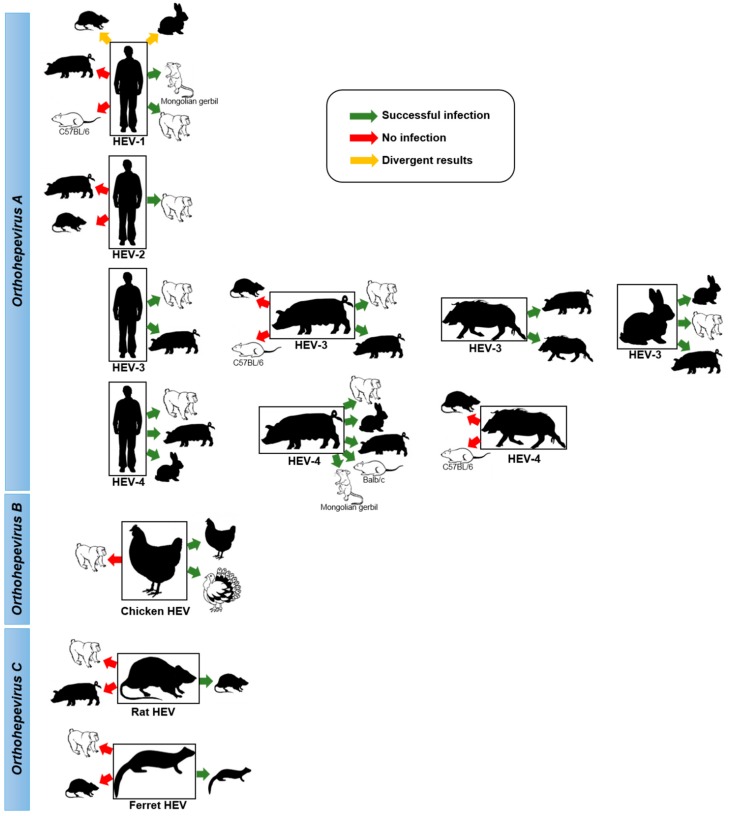
**Experimental inter-species transmissions of HEV.** Inter-species transmission of different HEV strains determined by the experimental infection of animal reservoirs (black silhouette) or animal models (white silhouette). Details and references of the different experiments presented are given in the text.

As described above, animal models and HEV natural reservoirs have been used to study experimentally the interspecies transmission of different HEV species and genotypes. The results obtained from these different studies are summarised in Figure 3. They clearly show that HEV-1 and HEV-2 are restricted to humans whereas HEV-3 and HEV-4 are naturally present in several animal species and can cross the species barrier. This difference raises the question of species barrier determinants. Several studies have suggested that genetic elements present in HEV ORF1 are involved in species barrier crossing [118,119,120,121].

In addition, the zoonotic transmission of HEV-3 and HEV-4 from swine, wild boar and deer to human via the consumption of contaminated meat has been proven. Molecular and phylogenetic analyses of HEV-3 and HEV-4 sequences from human and pig origin have shown high identity between the two populations and the absence of species clustering [49]. This suggests that swine HEV-3 and HEV-4 may not require any adaptation to jump between these two species. A study showing that the consensus sequence of HEV-3 is identical during transmission from human to swine is in agreement with this hypothesis [122]. However, it is still unclear whether strains of HEV-3 and HEV-4 present in other animals can cross the species barrier and infect humans. For example, the ability of rabbit HEV to infect humans and its contribution to zoonotic hepatitis E infection remain to be determined. The successful infection of cynomolgus macaques with rabbit HEV suggests that inter-species transmission of rabbit HEV-3 to human is possible [78]. The identification of a human strain that is closely related to rabbit strains is also in agreement with this hypothesis [51]. Further studies are also needed to determine the risk of zoonotic transmission of other strains from the *Orthohepevirus A* species such as wild boar HEV-5 and HEV-6. In addition, the recent identification of a human case associated with HEV-7 strongly suggests that this genotype is transmissible from camels to humans [54]. However, the contribution of HEV-7 to zoonotic hepatitis E remains to be clarified. Since zoonotic genotypes such as HEV-3 and HEV-4 can infect multiple animal species, it is also important to determine experimentally whether HEV-7 can infect other species such as rabbit and swine. This will help to clarify whether second or new reservoirs of this potential zoonotic genotype might exist or appear through direct transmission from camels or through human intervention.

## 5. Transmission Pathways of Zoonotic HEV

Since the discovery of swine HEV in 1997 [10], the risk of zoonotic transmission of HEV has been questioned and concern for public health has been raised. The first direct evidence of zoonotic transmission of HEV to humans was provided six years later following cases of HEV infection among patients who had consumed sashimi of Sika deer [123]. HEV-3 RNA was retrieved from the left-over deer meat and its sequence was found to be identical to those from the patients (326 nt within HEV ORF1). Three case reports have then provided additional direct evidence that HEV is a zoonosis that can be transmitted via the consumption of contaminated food. In these studies, identical or near identical HEV sequences were detected in patients suffering from hepatitis E and animal products they had consumed: grilled wild boar meat in Japan [124], pig meat in Spain [125] and ficatellu sausage from Corsica [126]. Several reports in Japan, France, Spain and Australia have also linked sporadic cases or outbreaks of hepatitis E with the consumption of raw or undercooked pork or wild boar products (meat, liver, liver paté, ficatellu or liver-based stuffing) without direct proof that these food items were the source of the infection [126,127,128,129,130,131,132,133]. These data are supported by studies showing that the consumption of pork and wild-boar meat and processed products is a risk factor for autochthonous HEV infection and HEV seropositivity [129,134,135,136,137,138]. In one of these studies, consumption of offal and wild-boar meat was found to be associated with autochthonous HEV infection in Germany [134]. Eating pork meat, pork liver sausages, game meat and offal was also found as a major contributor to the presence of anti-HEV antibodies in a recent nationwide survey performed in France [135].

HEV-3 and HEV-4 RNA is present throughout the pork food chain worldwide [130,133,139,140,141,142,143]. Studies have reported that 3%–11% of pig liver samples at the slaughterhouse are HEV positive in France (4%) [144], the Netherlands (6.5%) [145], Czech Republic (5%) [141], Italy (6%) [140], Spain (3%) [140], the United Kingdom (3%) [146], Japan (5%) [147] and the US (11%) [148]. HEV RNA was also found in the liver of wild boars (5.8%), deer (3.2%) and wild rabbits (5%) hunted in southwestern France [149] and in the liver of wild boars (1.9%) hunted in north-western Italy [150]. HEV RNA has also been detected in pork sausages sold in the UK (10%) [146] and in Spain (6%) [140]; in ficatelli (30%), dried salted liver (3%), quenelle and quenelle paste (25%) and dried or fresh liver sausages (29%) sold in France [139]; in raw and dry liver sausages purchased in Italy [141], in raw sausages (20%) and liver sausages (22%) sold in Germany [142]; and in pork pâté and blood sausage (36%) sold in Brazil [151].

Several studies have shown that such commercially-available pork livers and pork-derived products containing raw liver can contain infectious virus and are potential sources of foodborne HEV. Pigs inoculated intravenously with homogenates from contaminated pig livers sold in grocery stores in the United States became infected with HEV [148]. Moreover, HEV was successfully cultured in human cell lines inoculated with extracts from ficatelli sausages produced in France [152] or raw porcine liver purchased from grocery stores in Japan [153].

Recently, a liver-transplant patient from the Middle East who regularly consumed camel meat and milk was found to be infected with camelid HEV-7 [54]. Other animal reservoirs and genotypes of HEV might then be involved in the foodborne transmission of HEV. Milk from HEV-infected animals could also represent another source of zoonotic HEV that need to be further investigated.

Other foods that are not derived from animal products can be contaminated with HEV and are possible sources of foodborne HEV transmission. For instance, HEV-3 or HEV-4 RNA has been found in mussels from Galicia (14.81%) [154] and Scotland (85%) [155]; in oysters from coastal regions in Korea (8.7%) [156]; in bivalves from Japanese rivers [157]; and in shellfish from the coastal waters of China [158]. Moreover, experimental bioaccumulation has shown that oysters, flat oysters, mussels and clams can concentrate HEV, mostly in their digestive tissues [159]. Transmission of zoonotic HEV to human via the consumption of seafood has not been proven directly yet as no identical or near identical HEV sequences have been retrieved in patients suffering from hepatitis E and the seafood they had consumed. However, the consumption of shellfish has been strongly linked to an imported case of HEV-4 infection in a Japanese patient who travelled to Vietnam [160] and to an outbreak of hepatitis E on a cruise ship (HEV-3) [161].

In addition, HEV RNA has been found in strawberries in Canada [162], in frozen raspberries sold in Europe [163] and in the salad vegetable supply chain in Europe [164], suggesting that soft fruits and vegetables can also be contaminated with HEV RNA. A study has also suggested that herbs and spices can be contaminated with HEV (0.9%) [165].

The presence of HEV in shellfish, vegetable and fruits is likely caused by the contamination of surface and irrigation water with animal sewage. HEV RNA has been detected in swine sewage and manure worldwide [133,166,167,168,169] and it was shown that such waste products can be infectious when inoculated experimentally to pigs [169]. Runoff or insufficient treatment of sewage water from pig farms and use of manure as soil fertiliser could then lead to the contamination of neighbouring surface water. This hypothesis is supported by studies that found HEV RNA in surface water in proximity of pig farms [170,171]. HEV sequences similar to sequences found in patients with autochthonous hepatitis E infection and in swine have also been repeatedly found in river and seawater [172,173,174]. In addition, some of the shellfish found to be HEV RNA positive in Scotland were harvested near a slaughterhouse and pork processing plant [118].

The presence of HEV in food products derived from natural reservoirs of zoonotic HEV or food that are contaminated by surface and irrigation water raises concerns for public health and food safety worldwide. A subunit vaccine based on the expression of a truncated viral capsid is able to confer full protection after three doses and is licensed in China [175]. However, such vaccine has not yet been approved and commercialised in other countries. Prevention of zoonotic HEV relies mainly on avoiding raw and undercooked meat or selfish and cooking meat and meat products thoroughly. A few studies have been conducted using cell-culture [176,177] or in vivo swine experimental models [178,179] to determine the stability of HEV in the environment and in food products. Infectious viruses are still present in faecal suspension or cell-culture supernatant after heating at 56–60 °C [176,177]. Efficient inactivation of HEV in food products derived from infected pork liver was only achieved after a cooking time of at least 20 min at an internal temperature of 71 °C [178,179]. Temperatures equivalent to rare and medium-to-rare cooking are then insufficient to inactivate the virus and cooking food thoroughly and evenly is highly recommended to prevent foodborne transmission of HEV. Appropriate hygiene measures such as frequent hand and surface cleaning should also been followed when handling uncooked meat. In addition, swine waste should be properly eliminated and the use of swine manure as soil fertiliser should be regulated to reduce the risk of HEV contamination of crops and surface water.

As described above, HEV RNA has been detected in diverse food products ranging from meat and seafood, to fruits and vegetables. However, it is still unclear whether infectious lived virus can be present in most of these items. To solve this issue, a robust cell culture system needs to be developed rapidly. Such model will also help to clarify whether infectious viruses can be present in pork products that contain no liver such as cured ham. Indeed, the high HEV seroprevalence in human found in some countries such as France (22.4%) [135] cannot be explained only by the consumption of products containing raw pork liver such as ficatelli. Efforts are also needed to establish standardized methods to ensure a quality control of products at risk and the HEV oral-infectious dose remained to be determined to perform risk assessment studies.

In addition to foodborne routes of transmission, seroprevalence studies have suggested that direct contacts with infected animal reservoirs are risk factors for HEV exposure. Higher seroprevalence of anti-HEV antibodies was found in swine workers and veterinarians in the United States [180,181]; in swine veterinarians in France [137] and the Netherlands [145]; in swine farmers in Sweden [182], France [137] and Moldovia [183]; and in pork butchers in Burkina Faso [184]. In addition, higher HEV seroprevalence was detected among French hunters [100] and among forestry workers in France [137,185] and Germany [186]. The presence of stools from infected wild animals in forest may represent a source of contamination for this population. Interestingly, simple prevention measures such as wearing gloves and wearing boots for pig farmers, forestry workers or hunters are associated with reduced risk of HEV exposure [137,187]. A cross-sectional survey conducted in China has also found a higher anti-HEV IgG seroprevalence in seafood processing workers who have direct contacts with raw seafood [188]. Direct contacts with contaminated food and water might then represent a risk of HEV infection.

Contact with pet pig might also represent a possible source of HEV infection. In one study, frequent contact with a pet pig was reported to be the most likely cause of contamination of a French patient with acute hepatitis E [189].

## 6. Conclusions

Many novel strains of HEV have been identified in the last decades in diverse animal species. These discoveries have led to the reviewing of the Hepeviridae family classification and the ratification by the ICTV of a new taxonomic structure [8]. Nevertheless, it is very likely that additional HEV variants exist and that this classification will further evolve in the future. Available diagnostic tools are based on identified HEV strains and are probably not able to detect all the existing HEV strains. It is essential then that new molecular techniques are designed rapidly to detect a larger diversity of HEV strains and hosts. The wider use of metagenomics and deep sequencing could also contribute to the identification of HEV variants. Swine have been studied as the main HEV reservoir since its discovery in the late 1990s and HEV screenings in food have focussed mainly on pork-derived meat and meat products. However, it is possible that other animal reservoirs representing a significant risk for the zoonotic transmission of HEV exist. More studies are then clearly needed to screen a larger variety of food products derived from diverse animal species, including rabbit, camel and many others for the presence of HEV RNA and infectious virus. An exhaustive understanding of the extent of the animal reservoirs and transmission routes representing a risk for zoonotic hepatitis E infection is essential to prevent and control efficiently the disease in the future.
